# Innovative Nurse-Led Community Health Centre–Corrections Partnership for Hepatitis C Testing and Treatment in Victoria, British Columbia

**DOI:** 10.3390/v17121590

**Published:** 2025-12-06

**Authors:** Marion Selfridge, Tamara Barnett, Lesley Munro, Kiffer Card, Sarah Nishimura, Adam Beaumont, Catherine Clarke, Kellie Guarasci, Karen Lundgren, Katie Besko, Anne Drost, Chris Fraser

**Affiliations:** 1 Cool Aid Community Health Centre, Victoria, 713 Johnson Street, Victoria, BC V8W 1M8, Canada; tbarnett@coolaid.org (T.B.);; 2 Canadian Institute of Substance Use Research, University of Victoria, Health and Wellness Building, 2300 McKenzie Avenue, Victoria, BC V8N 5M8, Canada; 3 Department of Nursing, York University, 301A 4700 Keele Street, Toronto, ON M3J 1P3, Canada; 4 Vancouver Island Regional Correctional Centre, 4216 Wilkinson Road, Victoria, BC V8Z 5B2, Canada; 5 Faculty of Health Sciences, Simon Fraser University, 8888 University Drive SFU, Burnaby, BC V5A 1S6, Canada; 6 Victoria Native Friendship Centre, 231 Regina Avenue, Victoria, BC V8Z 1J6, Canada; 7 Pacific Centre Family Services Association, 324 Goldstream Ave, Victoria, BC V9B 2W3, Canada; 8 Department of Family Medicine, University of British Columbia, David Strangway Building, 5950 University Blvd, Vancouver, BC V6T 2A1, Canada

**Keywords:** prison, corrections, HCV, linkage to care

## Abstract

People who are incarcerated experience a high rate of hepatitis C (HCV) worldwide, and HCV micro-elimination in prisons is an effective strategy to support treatment. In Victoria, British Columbia, administrative barriers limited HCV testing and treatment at Vancouver Island Correctional Centre (VIRCC), and people who were HCV RNA+ were lost to follow up. Cool Aid Community Health Centre (CACHC) is an inner-city, primary care clinic that serves a marginalized population. The CACHC HCV nurse coordinator with the VIRCC nurse held HCV testing ‘blitzes’ at VIRCC and offered phlebotomy for screening and pre-treatment bloodwork. Clients who tested HCV RNA+ were started on HCV treatment and if discharged before completion, CACHC followed them in the community. A retrospective chart review was conducted to identify all clients who accessed HCV testing and treatment through the VIRCC partnership. To date, 230 clients were tested: 49 tested HCV antibody+, 11 tested HCV RNA+, and 10 started on treatment (6 SVR). Case management and consultation with the nurse coordinator and VIRCC nurse supported treatment starts for an additional 18 clients (14 SVR). This pragmatic and innovative approach to HCV care with people who are incarcerated demonstrated effective HCV testing and treatment. CACHC and VIRCC have established closer relationships and reduced barriers to reach and maintain continuity with this target population.

## 1. Introduction

Chronic hepatitis C virus (HCV) infection affects approximately 50 million people globally and can result in serious health complications and death if left untreated [1]. HCV is a global public health concern with 1 million new infections and approximately 242,000 HCV-related deaths occurring annually [1]. People who inject drugs (PWID) continue to make up the majority of new and existing HCV infections [2,3,4]. Globally, 26% of people incarcerated in prisons and 64% of incarcerated PWID have been exposed to HCV (HCV antibody positive) [5,6]. In Canada, approximately 76% of PWID have a history of incarceration [7] and people who are incarcerated are up to 40 times more likely to be exposed to HCV than the general population [8,9].

Novel interventions are necessary to minimize the risk of HCV acquisition for PWID, including decriminalizing drug use, minimizing unnecessary incarceration [7,10], and expanding harm reduction [11]. The World Health Organization (WHO) has called for a focus on prison settings to achieve global HCV elimination targets by 2030 [12,13] and has released updated guidelines recommending increased harm reduction, HCV testing, and simplified HCV treatment interventions, including a move towards a ‘one-stop-shop’ for prison settings [12]. However, access to HCV testing and treatment in prisons remains low compared to community settings and varies widely across countries, limiting opportunities to achieve elimination goals [7,8,14,15,16,17,18,19,20,21,22].

In Canada, federal prisons with sentences of two years and greater offer comprehensive HCV care, including opt-out screening at admission, direct-acting antiviral (DAA) treatment, and re-treatment options for non-responders or reinfected people who are incarcerated [23]. However, in provincial corrections, there is tremendous variability in HCV care practices within and across provinces [18]. These facilities are characterized by rapid intakes and releases because of the short duration of incarceration [17], averaging 28 days across Canada [24] and 65 days in British Columbia (BC) [25]. This makes it difficult to identify those with active HCV infection and to successfully complete an 8- or 12-week DAA regimen while incarcerated. For example, in BC correctional centres in 2021, 66% of those admitted were offered an HCV test, just 18% accepted HCV testing, and only 41% of those found to be HCV RNA positive received HCV treatment in custody [26]. Canadian correctional health staff identified several key barriers to HCV testing and treatment in 2020, including insufficient education for people who are incarcerated, inadequate screening strategies and protocols, limited resources and staff, restricted movement, poor continuity of care upon release, and gaps in provider knowledge and training related to HCV care [18].

Without ensuring continuity of care at the time of release, HCV treatments started in prison are often lost to follow up (LTFU) or disengaged from community care [8,27,28,29]. In Australia’s SToP-C project, 53% of participants who commenced treatment did not have documented treatment completion or sustained virological response (SVR), primarily due to prison transfer or release [30]. Individualized treatment plans and linkage to community care that is rooted in a multidisciplinary approach will likely increase treatment success [8,15].

Scaling up HCV testing and treatment in correctional settings, or “micro-elimination” approaches [31], has been shown to reduce HCV transmission among PWID, both in prisons and the community [20,32,33,34], and are cost-effective [33,35,36,37]. While this approach is advocated for widely [2,38], there is limited information on how to implement HCV testing and treatment that creates connections between a short stay correctional facility and community health care without requiring unsustainable clinical care pathways, including research interventions. This paper shares the HCV micro-elimination efforts and partnership of the Cool Aid Community Health Centre (CACHC) and the Vancouver Island Regional Correctional Centre (VIRCC) through testing blitzes, collaborative case management, communication, and investment in relationship-building to better serve people who are incarcerated in the Victoria, BC area. Nurses and other HCV clinicians from various communities regularly approach the second author, a community clinic HCV nurse coordinator, inquiring: “But how do you actually do this work? How do we get started?” While each community is diverse and requires specific interventions, we hope this paper provides pragmatic and specific details that support correctional health staff and community nurse-led HCV programs to begin or enhance their own efforts in HCV micro-elimination.

### 1.1. Local Context for HCV Testing and Treatment for People Who Are Incarcerated

The Cool Aid Community Health Centre (CACHC), located in Victoria, BC is an inner-city, interdisciplinary primary health care centre serving 7100 clients living with housing instability and chronic mental health and substance use challenges. The nurse-led HCV treatment program strives to provide trauma-informed, culturally safe, competent, flexible, low-barrier access to HCV screening, treatment, and follow-up. The program is integrated in primary care services using an equity-based, harm reduction framework [39,40,41]. On site and outreach sexually transmitted and blood borne infection (STBBI) testing has provided PWID and other key populations (i.e., sex workers, and gay and bisexual men who have sex with men [42]) with easy access to non-judgmental phlebotomy from nurses experienced in accessing veins compromised by injection drug use.

After successful HCV treatments within the clinic, the nurse-led HCV treatment team began a series of micro-elimination projects for HCV testing and treatment of clients in supported housing sites [43], pharmacies [44], COVID-19 sheltering sites [45], and outreach peer testing [46] to decrease the prevalence of HCV in the community. During the weekly case management appointments scheduled for all clients prescribed HCV treatment through the clinic, nurses would discover clients had disappeared from housing sites and stopped receiving HCV treatment from pharmacies. They had limited success determining their incarceration status due to confidentiality, even when expensive and life-changing HCV treatments were missed. After witnessing global successes in HCV testing and treatment in prisons, the CACHC HCV treatment team reached out to nearby Correctional Health Services (CHS) at VIRCC.

Locally, provincially incarcerated males (referred to as clients by VIRCC and throughout this paper) are sentenced to two years less a day at VIRCC. The Provincial Health Services Authority’s (PHSA) Correctional Health Services team assumed responsibilities in 2017 to provide continuity in health care, mental health support, and addiction services across BC. Whenever a client comes to a correctional centre, they are assessed by medical and mental health care staff as part of the admissions process [25]. At VIRCC, the Access and Transition (A&T) nurse reviews Hepatitis A, B, C, HIV, and Syphilis laboratory results on the provincial system.

Several administrative barriers have limited HCV testing and treatment at VIRCC. In the past, VIRCC clinical staff had limited information around current HCV treatments and were hesitant to start clients on treatment as their incarceration period may not last as long as HCV treatment. It has been challenging to routinely test for STBBIs, including HCV, as phlebotomy at VIRCC is only provided once a week in two-hour blocks by the local lab. This process requires clients to be brought from their unit to the health centre, which can take up to 25 min to move through the correctional centre. Often, there are over 200 clients at VIRCC, many with significant health, substance use, and mental health challenges [25]. However, only 6–9 blood draws are completed by the local lab each week. This has been a barrier to accessing HCV testing and the required HCV pre-treatment bloodwork as clients are prioritized for phlebotomy based on severity of their health conditions. Although other HCV research has demonstrated the efficacy of point-of-care RNA testing [30,47], currently a fibrosis assessment by APRI (AST to Platelet Ratio Index) or FIB-4 (fibrosis-4), using a non-invasive blood test before beginning treatment, is a requirement of reimbursement for provincial payment of HCV treatment. This requirement is based on a specific set of laboratory results that limits the efficacy of point-of-care testing locally and increases the number of steps required and length of time before HCV treatment can be initiated.

### 1.2. Community-Correctional Health Services Relationship Building

The CACHC HCV nurse coordinator reached out to the VIRCC health care team to create stronger connections between the organizations. VIRCC nurses were invited to attend local educational events that shared recent HCV-related research from international conferences. Several preceptorships were organized where VIRCC nurses spent the day at CACHC to learn about HCV case management approaches, current DAA regimes, and the specific details for successful applications for provincial reimbursement of DAA treatment. VIRCC staff became comfortable calling the CACHC HCV nurse coordinator for advice and clinical consultation. Prior to the partnership between CACHC and VIRCC, there was a lack of communication between clinical staff case managing HCV treatment that led to HCV RNA positive clients being LTFU. The preceptorships and long-term relationship building has improved VIRCC clinical staff knowledge around HCV treatment and created more open and collaborative case management of people who are incarcerated.

### 1.3. Establishing Testing Blitzes at VIRCC

The CACHC HCV treatment nurse and VIRCC health team identified that increased on-site STBBI testing, including HCV testing at VIRCC, would help facilitate HCV treatment starts. Due to the limited phlebotomy scope and equipment at VIRCC, the CACHC nurse offered regular visits to reduce wait times for testing. This began with discussions between the VIRCC warden and leadership to approve the project and develop policies. The CACHC HCV treatment nurse obtained the required security clearances and criminal record checks to enter VIRCC.

The first two testing events brought clients from their cells to the health centre for phlebotomy. However, there was poor turnout. Clients shared that some unit officers would announce “whoever wants to be tested for HCV can go to healthcare.” Clients said they were hesitant to “come down” due to HCV stigma and potential theft of belongings left behind. They may be “stuck” in a concrete holding cell in the health unit for long periods of time in a room without peers for socializing, food, television, easy access to a toilet, and books or paper for writing or colouring. If there is other movement at VIRCC or if there is a “code”, all clients are locked down and they can be held in the health unit for hours.

Instead, the CACHC HCV treatment nurse and VIRCC nurse (third author) saw the potential benefits of holding HCV testing ‘blitzes’ on VIRCC units by offering STBBI education, phlebotomy for STBBI screening, and HCV pre-treatment bloodwork. Consultation and buy-in from stakeholders were key. The team emailed the assistant deputy warden and checked in with corrections officers about the plan. To approve the blitzes, they required an additional correctional officer to accompany the two nurses to ensure all equipment (tourniquet, butterfly cannulas, vacutainers, etc.) was secured and to manage extra traffic and potential crowding. The blitzes were planned when clients had a “long lock up” where they were locked in their cell for several hours so that all the clients could be let into the common area to be tested together. To respect “prison culture”, the VIRCC nurse met with unit representatives (i.e., correctional client leadership) in advance, and only after they approved would she book the event.

Before each testing event, the VIRCC nurse posted signs in the designated units and reminded staff and clients often. She also notified them that there would be incentives for phlebotomy including candy, full size chocolate bars (ensuring no foil wrapping for safety), or water flavouring for people with diabetes, and decks of cards for each unit. These incentives were chosen by the clients as they are not available for purchase in the canteen. The nurses asked clients which table to use, since some are tied to specific social groups and are “off limits”. This gave clients control and showed respect for unit culture. Clients were offered testing in a very public space of the unit. No personal or health information, including HCV testing, treatment, or substance use history was asked to protect privacy. The VIRCC nurse confirmed the name of each client by pointing to the labels and asking the client if the label was correct before attaching it to the blood tube.

It was vital to create a workflow at VIRCC and CACHC for lab processing and result reporting. Initially, the CACHC HCV treatment nurse brought all equipment required and left with blood tubes still needing to be labelled and processed at CACHC. A medical chart was started for anyone who did not have one in the CACHC electronic medical record to receive and document results. Clients were also added to and flagged in the clinic HCV case management spreadsheet as part of the initiative. All tubes were labelled with client identifiers and specific tubes centrifuged and prepared for courier to lab accessions at the local hospital. This took hours after the blitzes, especially as the blitzes grew to over 30 clients tested within two hours. To reduce this workload, the VIRCC nurse advocated for a centrifuge, blood tubes, butterfly needles, vacutainer, and other equipment to be available on site. With these items in place, the team was able to centrifuge and label the tubes on site and send the bloodwork directly from VIRCC by taxi to the hospital laboratory accession department. Additionally, the local hospital requested 48 h’ notice for the testing blitz bloodwork, so the lab manager could schedule an extra technician to handle the increased volume. Clear communication across all systems was vital for success.

Lab results were reported to clinical staff at VIRCC and CACHC, and the health staff at VIRCC followed up with abnormal results. Clients were told that “no news is good news”. Abnormal results, including HCV RNA positives, were shared with clients through the VIRCC nurse, and treatment plans implemented including HCV treatment, STBBI care, and Hepatitis A/B vaccines. Clients were started on HCV treatment at VIRCC and if released prior to completion, they were followed in the community by CACHC. The VIRCC nurse applied for HCV treatment and the VIRCC physician prescribed treatment. The BC Centre for Disease Control (BCCDC) advocated for simplified provincial reimbursement for HCV treatment for clients (Plan Z) which reduced the lengthy bureaucratic processes required to demonstrate income history or co-payments and accelerated HCV treatments.

## 2. Materials and Methods

A retrospective chart review was conducted of all clients who were identified for HCV DAA treatment through the CACHC. All participants who initiated DAA therapy between 1 November 2014 (first availability of DAA therapy in British Columbia) and 31 December 2024 were eligible for inclusion in this larger study. The second author extracted the data into an Excel spreadsheet and the data were reviewed and cleaned by the first author. The data were anonymized at the point of extraction and transferred via a secure file transfer protocol. An anonymized secondary analysis was performed of this data set in R Studio (v.4.5.2) to generate descriptive statistics. For this specific analysis, all clients who accessed HCV testing and treatment through (and identified in the database as part of) the VIRCC partnership micro-elimination project were eligible for inclusion. Extracted variables were very limited and included HCV antibody and RNA results, and specific micro-elimination project and treatment history (if known). HCV antibody levels were determined by venous blood samples using the Abbott Architect Anti-HCV assay. HCV RNA levels used to determine SVR results were obtained from plasma samples analyzed locally with the Abbott Realtime Assay (lower limit of detection < 12 IU/mL). SVR was defined as an HCV RNA level below the limit of quantification 12 weeks after treatment completion among all participants who received at least one dose of DAA therapy [intent-to-treat (ITT) population]. If HCV RNA was not assessed at 12 weeks post-treatment, the result of the next documented HCV RNA assessment was used to calculate SVR. The anonymized secondary analysis of the retrospective chart review was approved by the harmonized research ethics process through the University of Victoria and Island Health (H25-01813) and was conducted according to the Declaration of Helsinki and International Conference on Harmonization Good Clinical Practice (ICH/GCP) guidelines.

## 3. Results

**Figure 1:**
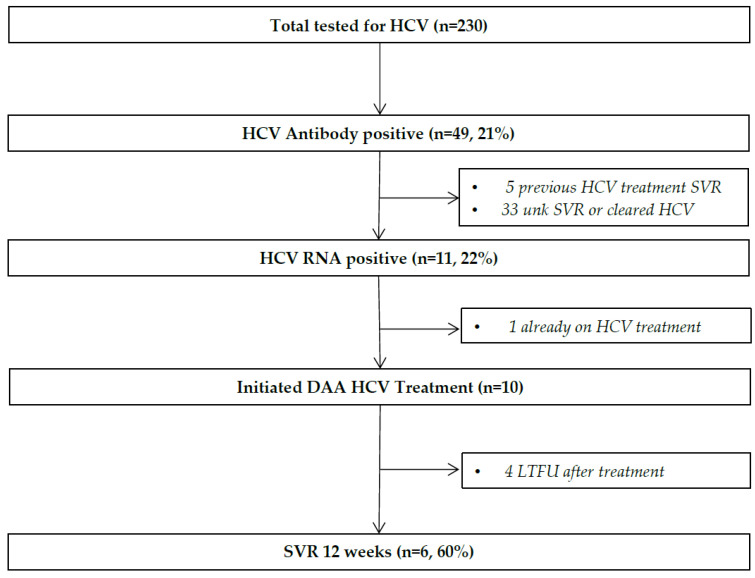
Description of Testing and Treatment for HCV Care Cascade of Correctional Population.

In total, 960 DAA HCV treatments have been started through CACHC from November 2014 to December 2024. Since the VIRCC partnership project started in March 2023 to July 2024, 10 onsite testing blitzes were conducted where 230 clients were screened by the CACHC HCV nurse coordinator at VIRCC. Of those tested, 49 clients have tested HCV antibody positive, 11 clients have tested RNA positive (including one already on treatment), and all 10 requiring therapy initiated and completed DAA treatment. Six (60% ITT) have achieved confirmed SVR (see Figure 1); the challenge of maintaining contact following release from VIRCC likely contributed to lower observed SVR rates.

Beyond the scheduled testing events, an additional 18 treatment initiations were identified and supported by both the CACHC HCV treatment nurse and the VIRCC nursing team as clients transitioned between corrections and community. Ten clients received treatment across both settings—eight achieved SVR, one requires SVR assessment but was subsequently LTFU, and one had not completed treatment at time of data analysis. Six clients were initially identified in the community and subsequently completed follow up and treatment while at VIRCC; five achieved SVR and one is LTFU. Two clients were identified and treated through the VIRCC program, with CACHC follow-up in the community for SVR blood work—one achieved SVR and one is LTFU. Of these 18 cases, four clients had a documented history of reinfection or treatment failure in the community. Identifying and initiating HCV treatment for people with prior treatment challenges during periods of incarceration may support HCV elimination efforts by reducing ongoing transmission risk and overcoming the barriers frequently encountered in community-based care.

### 3.1. Successes of the CACHC-Correctional Health Services Collaboration

Testing blitzes increased opportunities for testing and connection between correctional staff, health staff, and clients. Participation was higher when nurses went directly to units rather than calling clients down to the health unit. The most successful blitzes occurred outside during recreation time in the yard where huge queues formed. The busy blitzes may have cut phlebotomy wait time for the local lab by months. Both correctional staff and clients learned more about HCV and other STBBIs, which may have helped decrease stigma. Prior to and during blitzes, clients asked lots of questions: “we share razors and stuff, we have bleach and it is super watered down. Can we get hep C?” These interactions provided the nurses an opportunity to talk about other STBBIs, including syphilis which is increasing in the community. VIRCC correctional officers were also able to learn and ask questions through this process. Phlebotomy provided an opportunity to discuss vein health and harm reduction strategies. Providing testing and vaccines for hepatitis A and B created opportunities for other education about vaccines or anything else the clients asked about, including the concept of herd immunity within VIRCC.

The blitzes also appeared to reduce barriers between clients and the VIRCC health care team as the clients saw the nurse as someone who cared enough to come up to the units and put the effort in: “This matters so much to us that you came up to the unit and it’s so important because if we find out we have it, we can get treatment easily”. It also created opportunities for clients to learn more about CACHC services, especially the mobile health outreach vans that provide primary care at local shelters, as many clients knew they would be unhoused upon release. They were also informed of local HCV treatment and support by CACHC if discharged prior to completing treatment. This included linking daily dispensed Opioid Agonist Treatment (OAT) to their HCV treatment in the community to support adherence.

Partnerships have strengthened trust and capacity between organizations. Improved communication with VIRCC, CACHC, and provincial initiatives, including the BCCDC and provincial reimbursement strategies (Pharmacare), has expanded HCV treatment access. Rapid reimbursement through Plan Z has enabled clients without provincial coverage to access treatment. Community follow-up with CACHC supported successful HCV treatment completion and SVR testing for people cycling in and out of correctional centres, as intensive case management helps to address systemic barriers and maintain ongoing care.

### 3.2. Navigating Challenges

This project has had several challenges along the way. It has required continued buy-in and support from all levels at VIRCC; not just the warden, but the correctional officers on duty and the full health care team to continue effectively and persevere when problems occurred. A crucial reason for this was the extra time and work it took at each stage. Nursing staff at VIRCC and CACHC had to chart each client encounter. VIRCC nurses faced increased workload reviewing results, administering Hepatitis A and B vaccines, and abnormal result follow-up with already high workload levels. Like HCV treatment, it sometimes seemed inefficient to provide Hepatitis A and B vaccines when clients were unlikely to remain at VIRCC to complete the full vaccine series. However, the staff were encouraged to start the process. Working around correctional officers’ breaks, or reduced staff, and client counts during the long lockup period meant blood work often arrived at the hospital accessions in the evening when fewer lab technicians were working. Specific communication with lab accessions meant they would organize extra staffing and triage what could be processed the following day.

Phlebotomy at VIRCC during blitzes offered less control than in community settings. Clients could request the vein to be used, but it did not always work, and less preferred veins were used. Clients were not allowed to draw their own blood for safety reasons. For some, phlebotomy was triggering: “I wish you were putting something in versus taking blood out sometimes”. While blitzes provided an opportunity to meet with many clients quickly, the public spaces of the units provided very limited confidentiality. This meant personal questions about health history were not asked, and potentially unnecessary blood work was drawn. It also meant that certain clients, in more secure custody, were never offered phlebotomy during blitzes. While blitzes increased access to STBBI testing and identified people for treatment, they did not provide complete testing throughout the facility.

## 4. Discussion

People who are incarcerated have poor health outcomes compared to the general population in Canada [9]. Reducing their barriers to health care services can improve both individual and public health outcomes [48,49,50] and push against narratives that people who are incarcerated are considered less “deserving” of health care [51]. While system-wide change is needed to develop policy and resources for systematic HCV screening of all people who are incarcerated on admission [18,38] that are quick and accessible [19,52,53,54], this paper provides a pragmatic example of creating HCV screening and treatment micro-elimination with the current resources available that helps increase skills and connections locally.

Creating connections to the local community has implications in reducing LTFU for people who are incarcerated who are moved between correctional centres or discharged while on treatment. Mental health and substance use challenges make it difficult for people leaving VIRCC to follow up in the community and have been shown to be barriers to HCV care [9,14,27,28,55]. While our current results (only 60% SVR from testing blitzes) are consistent with the literature that shows release from custody during HCV therapy is associated with lower cure rates [28,29,30,47,56,57], we continue to connect back with people LTFU, as they access CACHC’s mobile health outreach van or outreach peer testing events, or are identified if re-admitted to VIRCC. By increasing VIRCC health staff awareness of HCV treatment, recognizing and leveraging the cyclical nature of incarceration [58] and improving communication, we have helped to reduce HCV treatment interruption [14].

Improved linkage to HCV treatment and other health care is vital [18,29,30] and should be supported with community-based services following release from prison [8,28,38,59]. Access to health care, housing, social services, and social support facilitates improved health after release from custody [50] and can help those who do not know where to begin or are overwhelmed or uncomfortable accessing services independently [59]. Active navigator programs, both for those who are continuing treatment and those still needing treatment, show great promise [14,47,58], although many still slip through the cracks as people move outside of the community where they were incarcerated or their phone numbers change [58,60].

The integration of people with lived and living experience of incarceration (peers) into corrections-based care has been associated with increased knowledge, reduced risks, and improved engagement with health care services by reducing stigma and fear, and increasing trust [29,61,62]. Linking people who are incarcerated to peer support and mentoring prior to and during release can support successful transition back into the community [53,63,64]. In BC, the peer-led Unlocking the Gates Services Society utilizes Peer Health Mentors to provide timely support at release and linkage to HCV care in some communities [60]. Providing traditional Indigenous medicine bundles [60] has been one way of acknowledging and supporting the vastly over-represented Indigenous populations in corrections, due to the ongoing impacts of colonization and residential school experience [61].

To optimize HCV elimination efforts in correctional settings, key stakeholders need to be engaged [15], including leadership, correctional and health staff, and people who are incarcerated who can either encourage or shut down initiatives [29]. People who are incarcerated often fear being stigmatized by correctional staff, health care workers, and their peers, leading many to avoid existing testing and treatment services [51,63]. Providing education to both staff and people who are incarcerated can increase limited HCV knowledge among PWID [65,66], and alleviate the stigma that some people experience while seeking HCV care in correctional settings [51,62,63,67]. Ongoing work is needed to address confidentiality and inadvertent disclosure of HCV status [67]. Having a dedicated nurse as point person both in corrections and in the community greatly improved the success of this project. However, staffing is always in jeopardy by competing health issues and staff shortages [51] and dedicated staff time is required to replicate this project in other sites.

### Limitations

The limitations of this study include its reliance on chart review methods, which have known limits to sensitivity and are prone to missing data. Due to the time constraints and the public testing space, very limited demographic, transmission risk, incarceration duration, and HCV treatment information is available on the population. Based on the limited number of events, clients tested and treated, and organizations involved in this initiative, these results may not be replicable in other settings. Unknown baseline HCV prevalence within the population limits the measurable impact of the initiative. All results should be interpreted with caution and warrant further study.

## 5. Conclusions

While many people who are incarcerated living with or at risk of HCV do not have access to timely testing and treatment, myriads of successful prison-based HCV projects and initiatives have emerged [14,28,47,51,55,56,57,68,69]. In Canada, advocacy is needed to ensure gaps in HCV care in provincial correctional centres are reduced, by adopting opt-out screening, removing eligibility restrictions, and increasing HCV education programs to staff and people who are incarcerated [18]. However, gaps exist to equip organizations to engage in advocacy [51]. Our hope is that by providing HCV education and awareness to correctional officers and health team staff, they will be better equipped to navigate the simplification and streamlining of care and to help address barriers for the scaleup of HCV testing and treatment [47].

This pragmatic and innovative approach to HCV care with people who are incarcerated demonstrated enhanced testing and treatment of HCV, both in and out of corrections, and could be adapted to work with other provincial correctional centres and community health providers. CACHC and the VIRCC health team have established closer relationships, and reduced barriers to reach and maintain continuity of care with people with a history of incarceration and others who remain untreated for HCV.

## Data Availability

The data presented in this study are available on request from the corresponding author due to small case counts to protect confidentiality.

## References

[1] World Health Organization (2025). Hepatitis C. https://www.who.int/news-room/fact-sheets/detail/hepatitis-c.

[2] Degenhardt L., Peacock A., Colledge S., Leung J., Grebely J., Vickerman P., Stone J., Cunningham E.B., Trickey A., Dumchev K. (2017). Global prevalence of injecting drug use and sociodemographic characteristics and prevalence of HIV, HBV, and HCV in people who inject drugs: A multistage systematic review. Lancet Global Health.

[3] Grebely J., Larney S., Peacock A., Colledge S., Leung J., Hickman M., Vickerman P., Blach S., Cunningham E.B., Dumchev K. (2019). Global, regional, and country-level estimates of hepatitis C infection among people who have recently injected drugs. Addiction.

[4] Spearman C., Dusheiko G., Hellard M., Sonderup M. (2019). Hepatitis C. Lancet.

[5] Larney S., Kopinski H., Beckwith C.G., Zaller N.D., Jarlais D.D., Hagan H., Rich J.D., van den Bergh B.J., Degenhardt L. (2013). Incidence and prevalence of hepatitis C in prisons and other closed settings: Results of a systematic review and meta-analysis. Hepatology.

[6] World Health Organization (2018). Guidelines for the Care and Treatment of Persons Diagnosed with Chronic Hepatitis C Virus Infection.

[7] Stone J., Fraser H., Lim A.G., Walker J.G., Ward Z., MacGregor L., Trickey A., Abbott S., Strathdee S.A., Abramovitz D. (2018). Incarceration history and risk of HIV and hepatitis C virus acquisition among people who inject drugs: A systematic review and meta-analysis. Lancet Infect. Dis..

[8] Kronfli N., Cox J. (2018). Care for people with hepatitis C in provincial and territorial prisons. CMAJ.

[9] Kouyoumdjian F., Schuler A., Matheson F.I., Hwang S.W. (2016). Health status of prisoners in Canada: Narrative review. Can. Fam. Physician.

[10] Dolan K., Wirtz A.L., Moazen B., Ndeffo-Mbah M., Galvani A., Kinner S.A., Courtney R., McKee M., Amon J.J., Maher L. (2016). Global burden of HIV, viral hepatitis, and tuberculosis in prisoners and detainees. Lancet.

[11] Lafferty L., Altice F.L., Leone F., Stoové M., Lloyd A.R., Hajarizadeh B., Kronfli N. (2024). Using nominal group technique with people who are incarcerated in Canadian federal prisons to identify barriers and solutions to improving Prison Needle Exchange Program uptake. Int. J. Drug Policy.

[12] World Health Organization (2019). Access to Hepatitis C Testing and Treatment for People Who Inject Drugs and People in Prisons: A Global Perspective: Policy Brief.

[13] World Health Organization (2021). The WHO Prison Health Framework: A Framework for Assessment of Prison Health System Performance.

[14] Akiyama M.J., Columbus D., MacDonald R., Jordan A.O., Schwartz J., Litwin A.H., Eckhardt B., Carmody E. (2019). Linkage to hepatitis C care after incarceration in jail: A prospective, single arm clinical trial. BMC Infect. Dis..

[15] Akiyama M.J., Kronfli N., Cabezas J., Sheehan Y., Thurairajah P.H., Lines R., Lloyd A.R. (2021). Hepatitis C elimination among people incarcerated in prisons: Challenges and recommendations for action within a health systems framework. Lancet Gastroenterol. Hepatol..

[16] Bartlett S.R., Buxton J., Palayew A., Picchio C.A., Janjua N.Z., Kronfli N. (2021). Hepatitis C Virus Prevalence, Screening, and Treatment Among People Who Are Incarcerated in Canada: Leaving No One Behind in the Direct-Acting Antiviral Era. Clin. Liver Dis..

[17] Bartlett S.R., Tiwana A., Dhillon D. (2022). Sexually transmitted and blood-borne infection testing and linkage to care during incarceration: There’s no time like the prison time!. UBC Med. J..

[18] Kronfli N., Dussault C., Bartlett S., Fuchs D., Kaita K., Harland K., Martin B., Whitten-Nagle C., Cox J. (2021). Disparities in hepatitis C care across Canadian provincial prisons: Implications for hepatitis C micro-elimination. Can. Liver J..

[19] Kronfli N., Dussault C., Chalifoux S., Kavoukian H., Klein M.B., Cox J. (2020). A randomized pilot study assessing the acceptability of rapid point-of-care hepatitis C virus (HCV) testing among male inmates in Montreal, Canada. Int. J. Drug Policy.

[20] Papaluca T., Hellard M.E., Thompson A.J., Lloyd A.R. (2019). Scale-up of hepatitis C treatment in prisons is key to national elimination. Med. J. Aust..

[21] Ruiz A.S., Fontaine G., Patey A.M., Grimshaw J.M., Presseau J., Cox J., Dussault C., Kronfli N. (2022). Identifying barriers and enablers to opt-out hepatitis C virus screening in provincial prisons in Quebec, Canada: A multilevel, multi-theory informed qualitative study with correctional and healthcare professional stakeholders. Int. J. Drug Policy.

[22] Winter R.J., Holmes J.A., Papaluca T.J., Thompson A.J. (2022). The importance of prisons in achieving hepatitis C elimination: Insights from the Australian experience. Viruses.

[23] Correctional Service Canada (2014). Infectious Disease Surveillance 2014 Hepatitis C Virus (HCV). https://www.csc-scc.gc.ca/publications/005007-3038-eng.shtml.

[24] Reitano J. (2017). Adult Correctional Statistics in Canada, 2015/2016.

[25] Province of British Columbia Profile of BC Corrections. British Columbia: 2020. https://www2.gov.bc.ca/assets/gov/law-crime-and-justice/criminal-justice/corrections/reports-publications/bc-corrections-profile.pdf.

[26] Bartlett S., Yu A., Young P., Korchinski M., Desrosiers N., Ang R., Esmail Z., Romm D., Luster D., Schmitz D. (2023). The first provincial correctional system-wide hepatitis C care cascade in Canada: Monitoring hepatitis C care in British Columbia provincial correctional centres. Can. Liver J..

[27] Aspinall E.J., Mitchell W., Schofield J., Cairns A., Lamond S., Bramley P., Peters S.E., Valerio H., Tomnay J., Goldberg D.J. (2016). A matched comparison study of hepatitis C treatment outcomes in the prison and community setting, and an analysis of the impact of prison release or transfer during therapy. J. Viral Hepat..

[28] Chan J., Schwartz J., Kaba F., Bocour A., Akiyama M.J., Hobstetter L., Rosner Z., Winters A., Yang P., MacDonald R. (2020). Outcomes of hepatitis C virus treatment in the New York City jail population: Successes and challenges facing scale up of care. Open Forum Infectious Diseases.

[29] Lafferty L., Rance J., Byrne M., Milat A., Dore G.J., Grebely J., Lloyd A.R., Treloar C., SToP-C Study Group (2022). “You need a designated officer”–Recommendations from correctional and justice health personnel for scaling up hepatitis C treatment-as-prevention in the prison setting. Int. J. Drug Policy.

[30] Ryan H., Dore G.J., Grebely J., Byrne M., Cunningham E.B., Martinello M., Lloyd A.R., Hajarizadeh B. (2024). Hepatitis C treatment outcome among people in prison: The SToP-C study. Liver Int..

[31] Lazarus J.V., Safreed-Harmon K., Thursz M.R., Dillon J.F., El-Sayed M.H., Elsharkawy A.M., Hatzakis A., Jadoul M., Prestileo T., Razavi H. (2018). The micro-elimination approach to eliminating hepatitis C: Strategic and operational considerations. Semin. Liver Dis..

[32] Girardin F., Hearmon N., Castro E., Negro F., Eddowes L., Gétaz L., Wolff H. (2019). Modelling the impact and cost-effectiveness of extended hepatitis C virus screening and treatment with direct-acting antivirals in a Swiss custodial setting. Clin. Infect. Dis..

[33] He T., Li K., Roberts M.S., Spaulding A.C., Ayer T., Grefenstette J.J., Chhatwal J. (2016). Prevention of hepatitis C by screening and treatment in US prisons. Ann. Intern. Med..

[34] Godin A., Kronfli N., Cox J., Alary M., Maheu-Giroux M. (2021). The role of prison-based interventions for hepatitis C virus (HCV) micro-elimination among people who inject drugs in Montréal, Canada. Int. J. Drug Policy.

[35] Dalgic O.O., Samur S., Spaulding A.C., Llerena S., Cobo C., Ayer T., Roberts M.S., Crespo J., Chhatwal J. (2019). Improved health outcomes from hepatitis C treatment scale-up in Spain’s prisons: A cost-effectiveness study. Sci. Rep..

[36] Cuadrado A., Llerena S., Cobo C., Pallás J.R., Mateo M., Cabezas J., Fortea J.I., Alvarez S., Pellón R., Crespo J. (2018). Microenvironment eradication of hepatitis C: A novel treatment paradigm. Off. J. Am. Coll. Gastroenterol.|ACG..

[37] Palmer A., Papaluca T., Stoové M., Winter R., Pedrana A., Hellard M., Wilson D., Thompson A., Scott N., Partnership E.V. (2021). A costing analysis of a state-wide, nurse-led hepatitis C treatment model in prison. Int. J. Drug Policy.

[38] Action Hepatitis Canada (2022). Prison Health is Public Health: The Right to Hepatitis C Prevention, Diagnosis, and Care in Canada’s Correctional Settings.

[39] Milne R., Price M., Wallace B., Drost A., Haigh-Gidora I., Nezil F.A., Fraser C. (2015). From principles to practice: Description of a novel equity-based HCV primary care treatment model for PWID. Int. J. Drug Policy.

[40] Selfridge M., Cunningham E.B., Milne R., Drost A., Barnett T., Lundgren K., Guarasci K., Grebely J., Fraser C. (2019). Direct-acting antiviral treatment for hepatitis C, reinfection and mortality among people attending an inner-city community health centre in Victoria, Canada. Int. J. Drug Policy.

[41] Selfridge M., Cunningham E.B., Barnett T., Drost A., Gray-Schleihauf C., Guarasci K., Lundgren K., Milne R., Grebely J., Fraser C. (2021). Reinfection following successful direct-acting antiviral therapy for HCV infection among people attending an inner-city community health centre in Victoria, Canada. Int. J. Drug Policy.

[42] Selfridge M., Card K.G., Lundgren K., Barnett T., Guarasci K., Drost A., Gray-Schleihauf C., Milne R., Degenhardt J., Stark A. (2020). Exploring nurse-led HIV pre-exposure prophylaxis in a community health care clinic. Public Health Nurs..

[43] Selfridge M., Barnett T., Lundgren K., Guarasci K., Milne R., Drost A., Fraser C. (2022). Treating people where they are: Nurse-led micro-elimination of hepatitis C in supported housing sites for networks of people who inject drugs in Victoria, Canada. Public Health Nurs..

[44] Selfridge M., Barnett T., Lundgren K., Guarasci K., Drost A., Fraser C. (2024). ‘I just never wanted them to feel uncomfortable’: Barriers to pharmacy-based identification and treatment of hepatitis C in Victoria, Canada. Can. Liver J..

[45] Selfridge M., Barnett T., Drost A., Guarasci K., Lundgren K., Roy H., Fraser C. Closing the loop: Ongoing nurse-led hepatitis C (HCV) micro-elimination projects in supportive housing sites and shelters for people who use drugs (PWUD) in Victoria, Canada. Proceedings of the Canadian Liver Meeting Conference.

[46] Selfridge M., Barnett T., Drayton D., Drost A., Gibbons T., Guarasci K., Lundgren K., Russell B., Stavely D., Fraser C. Working with people with lived experience of hepatitis C: Point of care testing in Victoria, British Columbia. Proceedings of the British Columbia Centre for Substance Use Conference.

[47] Sheehan Y., Cunningham E.B., Cochrane A., Byrne M., Brown T., McGrath C., Lafferty L., Tedla N., Dore G.J., Lloyd A.R. (2023). A ‘one-stop-shop’point-of-care hepatitis C RNA testing intervention to enhance treatment uptake in a reception prison: The PIVOT study. J. Hepatol..

[48] Wurcel A.G., Reyes J., Zubiago J., Koutoujian P.J., Burke D., Knox T.A., Concannon T., Wurcel A.G., Reyes J., Zubiago J. (2021). “I’m not gonna be able to do anything about it, then what’s the point?”: A broad group of stakeholders identify barriers and facilitators to HCV testing in a Massachusetts jail. PLoS ONE.

[49] John Howard Society of Ontario (2016). Fractured Care: Public Health Opportunities in Ontario’s Correctional Institutions.

[50] Hu C., Jurgutis J., Edwards D., O’Shea T., Regenstreif L., Bodkin C., Amster E., Kouyoumdjian F.G. (2020). “When you first walk out the gates… where do [you] go?”: Barriers and opportunities to achieving continuity of health care at the time of release from a provincial jail in Ontario. PLoS ONE.

[51] Walker S.J., Shrestha L.B., Lloyd A.R., Dawson O., Sheehan Y., Sheehan J., Maduka N.B., Cabezas J., Akiyama M.J., Kronfli N. (2024). Barriers and advocacy needs for hepatitis C services in prisons: Informing the prisons hepatitis C advocacy toolkit. Int. J. Drug Policy.

[52] Crespo J., Llerena S., Cobo C., Cabezas J., Cuadrado A. (2019). HCV management in the incarcerated population: How do we deliver on this important front?. Curr. Hepatol. Rep..

[53] Janssen P.A., Korchinski M., Desmarais S.L., Albert A.Y., Condello L.L., Buchanan M., Granger-Brown A., Ramsden V.R., Fels L., Buxton J.A. (2017). Factors that support successful transition to the community among women leaving prison in British Columbia: A prospective cohort study using participatory action research. Can. Med. Assoc. Open Access J..

[54] Gratrix J., Smyczek P., Bertholet L., Lee M.C., Pyne D., Woods D., Courtney K., Ahmed R. (2019). A cross-sectional evaluation of opt-in testing for sexually transmitted and blood-borne infections in three Canadian provincial correctional facilities: A missed opportunity for public health?. Int. J. Prison. Health.

[55] Werling K., Hunyady B., Makara M., Nemesi K., Horváth G., Schneider F., Enyedi J., Müller Z., Lesch M., Péterfi Z. (2022). Hepatitis C screening and treatment program in Hungarian prisons in the era of direct acting antiviral agents. Viruses.

[56] Papaluca T.J., Tambakis G., Iser D., Thompson A.J. (2019). Effective prison-based treatment and linkage to care after release. Lancet Infect. Dis..

[57] Eisen L., Mor Z., Madar M., Rabinovitch R., Dadon Y., Sheffer R., Kaliner E., Goldstein L. (2023). Hepatitis C virus elimination program among prison inmates, Israel. Emerg. Infect. Dis..

[58] Papaluca T., Craigie A., McDonald L., Edwards A., Winter R., Hoang A., Pappas A., Waldron A., McCoy K., Stoove M. (2022). Care navigation increases initiation of hepatitis C treatment after release from prison in a prospective randomized controlled trial: The C-LINK Study. Open Forum Infect. Dis..

[59] Palis H., Young P., Korchinski M., Wood S., Xavier J., Luk N., Mahil S., Bartlett S., Brown H., Salmon A. (2024). “Shared experience makes this all possible”: Documenting the guiding principles of peer-led services for people released from prison. BMC Public Health.

[60] Gale N., Tiwana A., Bartlett S.R. (2023). Test Link Call Project Impact Evaluation Report 2021–2022.

[61] Statistics Canada (2022). Adult and Youth Correctional Statistics, 2020/2021.

[62] Bagnall A.M., South J., Hulme C., Woodall J., Vinall-Collier K., Raine G., Kinsella K., Dixey R., Harris L., Wright N.M. (2015). A systematic review of the effectiveness and cost-effectiveness of peer education and peer support in prisons. BMC Public Health.

[63] Crowley D., Murtagh R., Cullen W., Keevans M., Laird E., McHugh T., McKiernan S., Miggin S.J., O’Connor E., O’Reilly D. (2019). Evaluating peer-supported screening as a hepatitis C case-finding model in prisoners. Harm Reduct. J..

[64] McLeod K.E., Korchinski M., Young P., Milkovich T., Hemingway C., DeGroot M., Condello L.L., Fels L., Buxton J.A., Janssen P.A. (2020). Supporting women leaving prison through peer health mentoring: A participatory health research study. Can. Med. Assoc. Open Access J..

[65] Treloar C., Hull P., Dore G.J., Grebely J. (2012). Knowledge and barriers associated with assessment and treatment for hepatitis C virus infection among people who inject drugs. Drug Alcohol Rev..

[66] Childs E., Assoumou S.A., Biello K.B., Biancarelli D.L., Drainoni M.L., Edeza A., Salhaney P., Mimiaga M.J., Bazzi A.R. (2019). Evidence-based and guideline-concurrent responses to narratives deferring HCV treatment among people who inject drugs. Harm Reduct. J..

[67] Rance J., Lafferty L., Treloar C. (2020). ‘Behind closed doors, no one sees, no one knows’: Hepatitis C, stigma and treatment-as-prevention in prison. Crit. Public Health.

[68] Lafferty L., Sheehan Y., Cochrane A., Grebely J., Lloyd A.R., Treloar C. (2023). Reducing barriers to the hepatitis C care cascade in prison via point-of-care RNA testing: A qualitative exploration of men in prison using an integrated framework. Addiction.

[69] Kronfli N., Mambro A., Riback L.R., Ortiz-Paredes D., Dussault C., Chalifoux S., Del Balso L., Petropoulos A., Lim M., Halavrezos A. (2024). Perceived patient navigator services and characteristics to address barriers to linkage to hepatitis C care among people released from provincial prison in Quebec, Canada. Int. J. Drug Policy.

